# Supplementary material for: The relationship between serum eosinophil peroxidase and major basic protein levels in relation to severity and response to H1 antihistamines in chronic spontaneous Urticaria
Source: PLoS One. 2025 Nov 11;20(11):e0336118. doi: 10.1371/journal.pone.0336118 (PMC12604763; doi:10.1371/journal.pone.0336118)
Supplement: S1 Table — (ASST: autologous serum skin test; CRP: C-reactive protein; CSU: chronic spontaneous urticaria; IgE: immunoglobulin E; IgG: immunoglobulin G; TPO: Thyroid Peroxidase; UAS7: urticaria activity score over 7 days; *Man-Whitney U test,** χ2 test, ***Fisher Exact test, +T-test). (DOCX) [file pone.0336118.s001.docx]

**S1 Table. Characteristics of the study subjects**

| **Variables** | **Severe CSU (n = 60)** | **Non-severe CSU (n=60)** | **P value** |
| --- | --- | --- | --- |
| **Age (yr)** | 40.1±15.2 | 38.2±15 | 0.49^+^ |
| **Female** | 33 (55%) | 40 (66.7%) | 0.19** |
| **UAS7 (0-42)** | 32.4± 5.3 | 20 (14–23.5) | 0.000* |
| **Urticaria duration (weeks).** | 20 (12.4–52) | 19.5 (11.5–29) | 0.31* |
| **ASST (+)** | 34 (56.7%) | 36 (61%) | 0.63** |
| **Angioedema** | 25 (41.7%) | 19 (31.7%) | 0.26** |
| **CRP (mg/L)** | 1.8 [0.7–3.8] | 0.9 [0.4–2.3] | 0.02* |
| **Elevated CRP (>5mg/L)** | 8 (13.3%) | 6 (10%) | 0.57** |
| **Total IgE (IU/mL)** | 169.4 [130.5–331.1] | 166.5 [83.2–347.6] | 0.21* |
| **Elevated IgE (>100 IU/mL)** | 49 (81.7%) | 42 (70%) | 0.33*** |
| **IgG anti-TPO (kU/L)** | 0.34 [0.1–0.99] | 0.42 [0.14–0.98] | 0.75* |
| **Elevated IgG anti-TPO (≥34kU/L)** | 3 (5%) | 2 (3.3%) | 1*** |
| **Eosinophils (cells/L)** | 0.085 [0.044–0.169] | 0.11 [0.056–0.212] | 0.19* |
| **Eosinopenia (<0.05 x 10^9^/L)** | 17 (28.3%) | 13 (21.7%) | 0.40** |
| **Basophils (cells/L)** | 0.032 [0.021–0.045] | 0.035 [0.025–0.054] | 0.29* |
| **Basopenia (<0.01 x 10^9^/L)** | 1 (1.7%) | 0 (0%) | 1.00*** |

(ASST: autologous serum skin test; CRP: C-reactive protein; CSU: chronic spontaneous urticaria; IgE: immunoglobulin E; IgG: immunoglobulin G; TPO: Thyroid Peroxidase; UAS7: urticaria activity score over 7 days; *Man-Whitney U test,** χ^2^ test, ***Fisher Exact test, ^+^T-test)
