# Supplementary material for: The relationship between serum eosinophil peroxidase and major basic protein levels in relation to severity and response to H1 antihistamines in chronic spontaneous Urticaria
Source: PLoS One. 2025 Nov 11;20(11):e0336118. doi: 10.1371/journal.pone.0336118 (PMC12604763; doi:10.1371/journal.pone.0336118)
Supplement: S2 Table — (ASST: autologous serum skin test; CRP: C-reactive protein; CSU: chronic spontaneous urticaria; EPO: Eosinophil Peroxidase; IgE: immunoglobulin E; IgG: immunoglobulin G; MBP: Major Basic Protein; TPO: Thyroid Peroxidase; *Mann-Whitney U test, ++Kruskal-Wallis test). (DOCX) [file pone.0336118.s002.docx]

**S2 Table. Serum EPO and MBP concentrations in CSU according to some clinical/paraclinical characteristics**

| **Variables** | | **Spearman’s rho** | | **P value** | |
| --- | --- | --- | --- | --- | --- |
|  | | **EPO** | **MBP** | **EPO** | **MBP** |
| **Age (years)** | | –0.22 | 0.07 | **0.02** | 0.42 |
| **Urticaria duration (weeks)** | | 0.058 | –0.13 | 0.53 | 0.16 |
| **Eosinophils (cells/L)** | | 0.13 | –0.04 | 0.16 | 0.7 |
| **CRP (mg/L)** | | –0.14 | 0.1 | 0.12 | 0.3 |
| **Total IgE (IU/mL)** | | –0.07 | 0.12 | 0.47 | 0.2 |
| **IgG anti-TPO (kU/L)** | | 0.19 | –0.43 | **0.04** | **<0.001** |
|  | | **EPO (ng/mL)** | **MBP (ng/mL)** | **EPO** | **MBP** |
|  |  | *Median (interquartile range) or*  *mean ± standard deviation* | |  |  |
| **Sex** | **Male**  **(n = 47)** | 34.55 (22.31–42.73) | 362.87 (211.21–696.04) | 0.51* | 0.13* |
|  | **Female**  **(n = 73)** | 30.87 (21.94–36.89) | 331.32 (200.49–487.03) |  |  |
| **Angioedema** | **Positive**  **(n = 44)** | 30.62 (22.31–37.11) | 355.43 (233.52–489.59) | 0.90* | 0.78* |
|  | **Negative**  **(n = 76)** | 33.51 (20.61–38.26) | 322.15 (198.32-564.36) |  |  |
| **ASST** | **Positive**  **(n = 70)** | 34.45 (21.94–46.24) | 350.36 (212.65–534.2) | 0.08* | 0.78* |
|  | **Negative**  **(n = 50)** | 26.44 (21.94–36.34) | 326.27 (188.74–534.2) |  |  |
| **Eosinophils** | **Eosinopenia**  **(n = 30)** | 25.88 (18.9–35.94) | 449.11±272.67 | 0.09* | 0.47* |
|  | **Non-Eosinopenia (n = 90)** | 33.51 (22.31–40.97) | 322.15 (210.77–510.83) |  |  |
| **CRP** | **Elevated**  **(n = 14)** | 25.13 (21.94–36,89) | 445.02±282.47 | 0.54* | 0.58* |
|  | **Normal**  **(n = 106)** | 33.3 (21.94–37.62) | 339.5 (206.96–524.57) |  |  |
| **Total IgE** | **Low**  **(n = 5) (<40 IU/mL)** | 35.94 (17.37–36.34) | 196,15 (189.07–311.4) | 0.92^++^ | 0.13^++^ |
|  | **Normal**  **(n = 24)** | 31.98 (20.8–44.78) | 354.14±159.07 |  |  |
|  | **Elevated**  **(n = 91)** | 32.33 (22.31–37.4) | 263,05 (210.77–578.23) |  |  |
| **IgG anti-TPO** | **Normal**  **(n = 115)** | 31.83 (21.94–37.34) | 341.15 (211.21–534.2) | 0.35* | 0.41* |
|  | **Elevated**  **(n = 5)** | 34.55 (33.42–35.09) | 178.86 (171.24–595.25) |  |  |

(ASST: autologous serum skin test; CRP: C-reactive protein; CSU: chronic spontaneous urticaria; EPO: Eosinophil Peroxidase; IgE: immunoglobulin E; IgG: immunoglobulin G; MBP: Major Basic Protein; TPO: Thyroid Peroxidase; *Mann-Whitney U test, ^++^Kruskal-Wallis test)
